# Thermal Analysis of Stomatal Response under Salinity and High Light

**DOI:** 10.3390/ijms22094663

**Published:** 2021-04-28

**Authors:** Aleksandra Orzechowska, Martin Trtílek, Krzysztof Michał Tokarz, Renata Szymańska, Ewa Niewiadomska, Piotr Rozpądek, Katarzyna Wątor

**Affiliations:** 1 Faculty of Physics and Applied Computer Science, AGH University of Science and Technology, Al. Mickiewicza 30, 30-059 Kraków, Poland; Renata.Szymanska@fis.agh.edu.pl; 2 Photon Systems Instruments, Drásov 470, 664 24 Drásov, Czech Republic; martin@psi.cz; 3 Department of Botany, Physiology and Plant Protection, Faculty of Biotechnology and Horticulture, University of Agriculture in Kraków, Al. 29 Listopada 54, 31-425 Kraków, Poland; krzysztof.tokarz@urk.edu.pl; 4 The F. Górski Institute of Plant Physiology, Polish Academy of Sciences, Niezapominajek 21, 30-239 Kraków, Poland; e.niewiadomska@ifr-pan.edu.pl; 5 Malopolska Centre of Biotechnology, Jagiellonian University, Gronostajowa 7a, 30-387 Kraków, Poland; piotr.rozpadek@uj.edu.pl; 6 Department of Hydrogeology and Engineering Geology, Faculty of Geology, Geophysics and Environmental Protection, AGH University of Science and Technology, Al. Mickiewicza 30, 30-059 Kraków, Poland; wator@agh.edu.pl

**Keywords:** infrared thermal imaging, salinity, excessive light, light-induced temperature kinetics, stomatal conductance, evapotranspiration, photosystem II efficiency

## Abstract

A non-destructive thermal imaging method was used to study the stomatal response of salt-treated *Arabidopsis thaliana* plants to excessive light. The plants were exposed to different levels of salt concentrations (0, 75, 150, and 220 mM NaCl). Time-dependent thermograms showed the changes in the temperature distribution over the lamina and provided new insights into the acute light-induced temporary response of *Arabidopsis* under short-term salinity. The initial response of plants, which was associated with stomatal aperture, revealed an exponential growth in temperature kinetics. Using a single-exponential function, we estimated the time constants of thermal courses of plants exposed to acute high light. The saline-induced impairment in stomatal movement caused the reduced stomatal conductance and transpiration rate. Limited transpiration of NaCl-treated plants resulted in an increased rosette temperature and decreased thermal time constants as compared to the controls. The net CO_2_ assimilation rate decreased for plants exposed to 220 mM NaCl; in the case of 75 mM NaCl treatment, an increase was observed. A significant decline in the maximal quantum yield of photosystem II under excessive light was noticeable for the control and NaCl-treated plants. This study provides evidence that thermal imaging as a highly sensitive technique may be useful for analyzing the stomatal aperture and movement under dynamic environmental conditions.

## 1. Introduction

Infrared thermography (IR thermography) has been widely used in many fields of science and technology, including agriculture, horticulture, and plant physiology, as a non-destructive method for the detection of heat transfer, heat loss, hot spots, and temperature distribution [1,2,3,4,5]. Thermography is appropriate for studies of the effects of temperature on plants and has distinct advantages for the quantitative analysis of spatial and dynamic physiological information [1]. Leaf temperature varies with transpiration rate [6], which is largely a function of stomatal conductance and water vapour deficit. In well-watered plants, transpiration represents one of the most effective means of cooling because the specific heat capacity of water is higher than that of any other common substances [7,8]. Opening of the stomata results in evaporative cooling and a decrease in leaf temperature. The internal metabolic processes of plant tissues, as well as radiation absorption, can increase leaf temperature, whereas the latent heat of evaporation, heat conduction, and heat convection work to counteract such heating [9]. Thermal imaging has already been used for measuring stomatal conductance [10,11], detecting water stress [12,13], diseases and infections in plants [14,15,16], frost-sensitive species [17], mutations [18,19], and thermogenesis [20]. This technique has been used for screening salt tolerance in plants [21,22,23] and also for diagnosing the effects of salinity in a soilless culture [24], which caused a reduction in water content in plant tissues and stomata conductance. 

Salinity is a significant limiting factor for cereal production in many parts of the world. Research on salt-induced damage and physiological effects on crops has been reviewed extensively over the last few years. Sirault et al. [25] screened salt-tolerant wheat and barley using thermal image analysis. Furbank and Tester [26] suggested that thermal imaging could be applied to high-throughput phenotypic screening to reduce screening time and costs and to improve crop breeding. Urrestarazu [24] used IR thermography in the detection of salt stress in ornamental crops. Salinity is one of the abiotic stress factors that affects plant cell metabolism and reduces plant productivity. It impairs plant growth and development via water stress, cytotoxic-dependent accumulation of toxic ions such as sodium (Na^+^) and chloride (Cl^−^), and generation of reactive oxygen species (ROS) [27]. Salinity reduces osmotic water uptake and adversely influences carbon assimilation by reducing the photosynthetic rate, transpiration rate, stomatal conductance, and by degrading of chlorophyll (Chl) [28]. High levels of salt in the soil can disrupt the osmotic balance in the rhizosphere, making water less available for plants [29]. Salinity creates both, osmotic and ionic stress. Osmotic stress takes place within minutes to days and causes stomatal closure and a significant reduction in the rate of shoot growth. Decreases in transpiration rate, stomatal conductance, and leaf gas exchange are among the first responses to soil salinization [30,31,32]. A second, ionic-specific phase of a plant’s response to salinity takes place over days or even weeks and starts when salt accumulates to toxic concentrations in older leaves. This leads to a slowing down of metabolic processes, premature senescence, and finally cell death [29]. 

Under field conditions, plants are exposed to joint stress factors, where salt stress is most often combined with high light (HL). There have been many reports showing the simultaneous effects of salt stress and HL on plants [33,34,35]. Excess light decreases the photosynthetic electron transport chain activity. Under intense illumination, photosystem II (PSII) undergoes photoinhibition [36]. This phenomenon is triggered by ROS, which directly inactivate the photochemical reaction center of PSII [37,38]. Experimental evidence suggests that salt stress might enhance photodamage to PSII [39], but there is also a possibility that it may inhibit photodamage repair. The crucial factors that impact on plant response to light and salinity are the intensity of the stress and its duration [32]. Salinity also influences water status in plants and water is known to be a primary source of infrared absorption in plant tissue [40]. Plants exposed to salinity close their stomata to protect against water loss, which causes an increase in leaf temperature. 

Salinity-generating stress results in stomatal regulation, an important strategy that enables plants to cope with NaCl-induced osmotic and ionic stresses. Combined, movements of stomata are regulated through a set of complex processes, that have not been fully identified yet. However, those processes seem to converge and may result from hydropassive mechanisms (direct evaporation of water without metabolic involvement) [41] as well as morphological, physiological, and molecular metabolic-dependent mechanisms [42]. The latter comprises regulation of K^+^ transport, expression and activity of aquaporins [43], the signaling of ROS [44], guard cell calcium status, membrane fluidity, and phototropin activation [45]. Most of these processes are under the control of hormones [46]. Among them, stomata closure and opening seem to be mostly abscisic acid (ABA)-regulated. ABA-mediated stomata movements are associated with many different mechanisms, i.e., the activation of transcription factors mediated by protein kinases, receptor-dependent breakdown of starch into sugar and sugar-derived osmolytes, or the regulation of ABA-responsive element binding genes and proteins, which are directly or indirectly involved in stomatal closure [41,42,46]. 

This research investigates the potential of IR thermography for studying the response of *Arabidopsis thaliana* (*A. thaliana*) to salinity as well as the combination of salt stress and an acute HL. *A. thaliana* is less tolerant to salinity compared with other species under similar conditions of light and humidity [29]. Our goal was to visualize the kinetics of rapid changes in HL-induced stomatal opening in this model plant. To date, the use of thermal imaging for studying abiotic stresses such as salinity or HL has been limited to steady-state environmental conditions. To the best of our knowledge, ours is the first report to show the use of thermal imaging in the study of plants exposed to salinity under dynamic conditions i.e., the HL. This research shows the feasibility of the non-destructive imaging method in evaluating the short-term salinity and an acute HL treatment.

## 2. Results

### 2.1. Effect of Salinity on Dry Mass, Water Content, and Na^+^ Uptake by Plants

**Figure 1:**
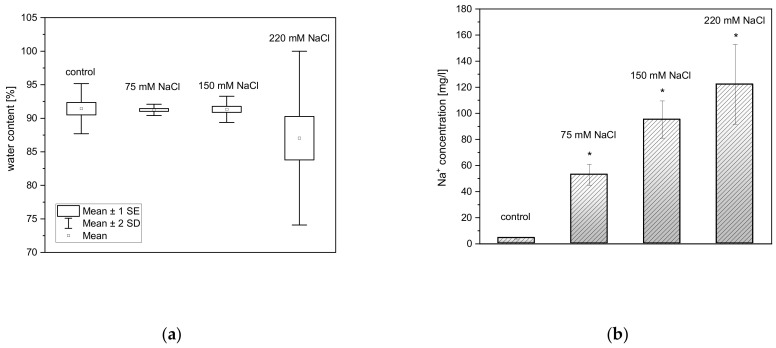
Water content of four-old-week rosettes of non- and salt-treated *A. thaliana* plants. Results are presented as box-and-whisker plots, showing mean and standard error at the 95% confidence level (**a**). Concentration of sodium ions accumulated in *A. thaliana* leaf rosettes after one-time treatment with 0, 75, 150, and 220 mM NaCl (**b**). Asterisks (*) indicate significant differences in Na^+^ content between non- and salt-treated plants (ANOVA, *p* ≤ 0.05).

Water is the first target of infrared absorption in plant tissues. For that reason, dry mass and water content of all the plants were estimated. NaCl exposure did not significantly affect the water and the dry mass content in rosette leaves of *A. thaliana* as compared to controls (Figure 1a).

To confirm that NaCl-treated plants absorbed the salt from the soil and distributed it within the plant, we carried out an ICP-OES analysis. Rosette leaves of *A. thaliana* had a sodium cations concentration ranging from 4.23 ± 0.63 mg/L to 122.23 ± 30.54 mg/L, which increased in a NaCl concentration-related manner (Figure 1b).

### 2.2. Effect of Salinity on Photosynthetic Pigment Content

**Figure 2:**
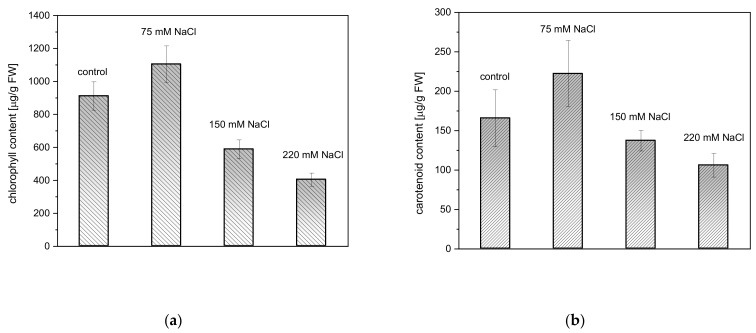
Total chlorophyll (**a**) and carotenoid (**b**) content in rosette leaves of four-week old *A. thaliana* plants, three days after exposure to salinity. Tests of statistical significance in Chl and Car content between the salt-treated plants compared to the controls were performed using ANOVA (*p* ≤ 0.05).

Salinity stress induced by 75, 150, and 220 mM NaCl had no clear negative influence on the content of photosynthetic pigments in *A. thaliana* rosette leaves (Figure 2). Carotenoid (Car) and chlorophyll (Chl) content in the salt-treated plants did not reveal significant differences as compared to the controls.

### 2.3. Gas Exchange and Fluorescence Measurements

**Figure 3:**
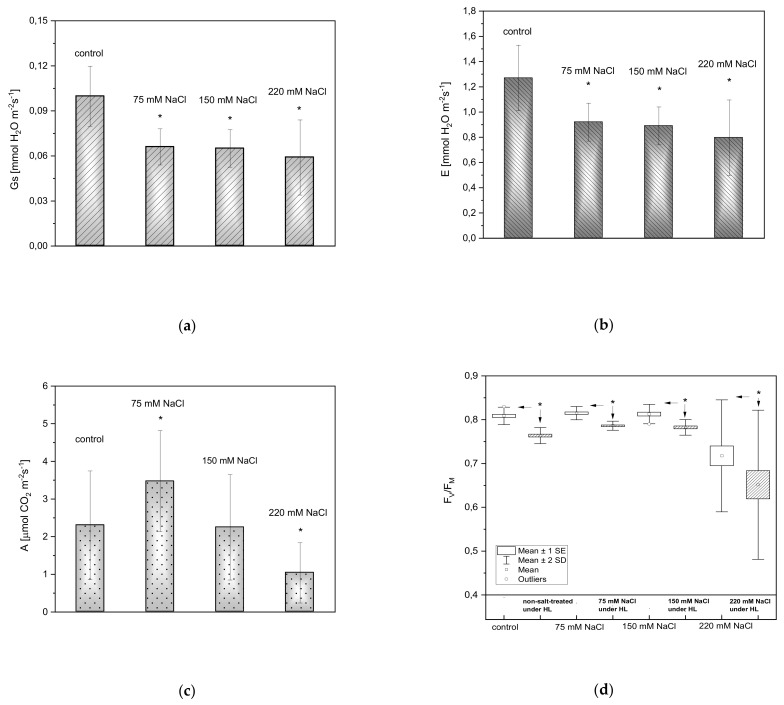
Stomatal conductance (**a**), transpiration rate (**b**), and the net CO_2_ assimilation rate (**c**) measured three days after inducing salt stress in 4-week old *A. thaliana* rosettes. Maximal PSII efficiency (F_V_/F_M_) in *A. thaliana* plants exposed to salinity under LL (empty box charts) and an acute HL (pattern-filled box charts) is shown in (**d**). Results are presented as box-and-whisker plots, showing mean, standard error, and outliers at the 95% confidence level. An asterisk (*) indicates significant differences (*p* ≤ 0.05, the Mann-Whitney *U* test) between control and salt-treated plants (**a**–**c**), and plants before (LL) and after an acute HL treatment (**d**).

Stomatal conductance (*Gs*), transpiration (*E*), and photosynthesis rate (*A*) are all parameters affected by salt stress. Stomatal conductance (Figure 3a) and transpiration rate (Figure 3b) decreased in plants exposed to salinity. Measurements of carbon dioxide uptake showed that the photosynthesis rate decreased in plants exposed to 150 and 220 mM NaCl (Figure 3c). Interestingly, in the case of plants treated with 75 mM NaCl, the photosynthetic rate was greater than in the controls. The maximum efficiency of PSII photochemistry was measured to estimate the influence of salinity on the activity of the photosynthetic apparatus in *A. thaliana*. Three days after the NaCl treatments, F_V_/F_M_ significantly decreased only in plants exposed to 220 mM NaCl (Figure 3d). Clear progressive decreases in F_V_/F_M_ occurred with increasing salt concentration under excessive light. In all plants exposed to HL, the reduction in F_V_/F_M_ was statistically significant.

### 2.4. Thermometric Measurements

**Figure 4:**
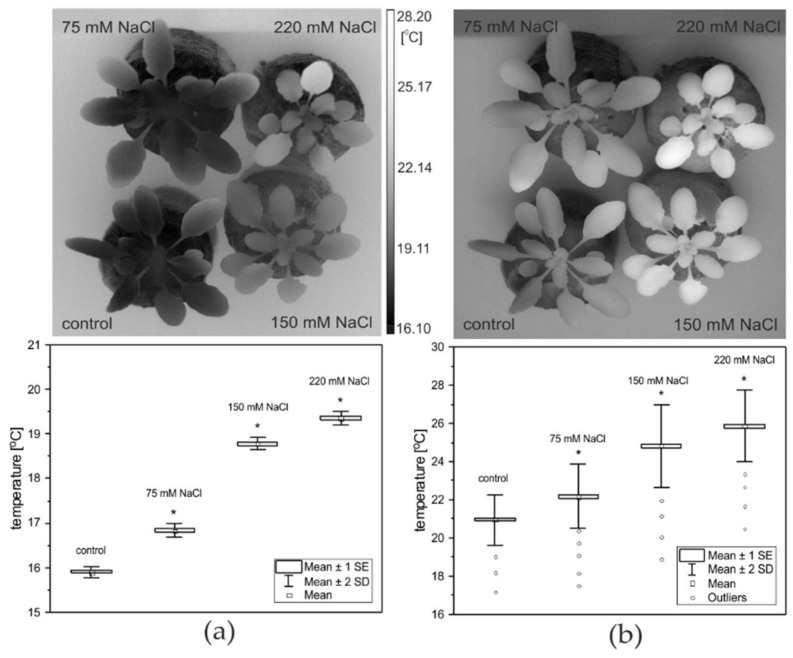
Infrared thermograms of LL-growing (**a**) and HL-treated (**b**) *A. thaliana* plants acquired three days after exposure to salinity (*upper panel*), and the average temperature distribution over the leaf rosettes of control and salt-treated plants (*lower panel*). Data are presented as box-and-whisker plots showing mean, standard error, and the outliers at the 95% confidence level. An asterisk (*) indicates significant differences (*p* ≤ 0.05, ANOVA test) between the non- and salt-treated plants before (LL) and after an acute HL treatment.

Thermal imaging was used to study the primary response of *A. thaliana* plants to salinity under acute HL. Figure 4 shows an exemplary representative thermographic image, taken at the same time from salt-treated plants juxtaposed with non-treated controls, which was measured just before (Figure 4a) and after (Figure 4b) the HL light onset.

The thermograms (TGs) presented in Figure 4a show the temperature changes over the lamina of plants exposed to salinity compared to the controls. The highest temperature is observed for the highest concentration applied (220 mM NaCl); however, this effect is enhanced under the HL treatment (Figure 4b). The average temperature distribution over the leaf rosettes of control and salt-treated plants under LL, and an acute HL, are shown in lower panels of Figure 4. Mild salt stress (75 mM NaCl) caused a difference in leaf temperature of 0.92 ± 0.02 °C, and 1.22 ± 0.02 °C under the HL concerning controls, whereas more significant differences were observed for higher NaCl treatments. Plants exposed to 150 mM NaCl revealed a temperature higher by 2.87 ± 0.02 °C (and 3.80 ± 0.02 °C under HL) in comparison to the controls. The largest and most significant increase in temperature occurred in plants treated with 220 mM NaCl. These plants showed a temperature that was greater by 3.45 ± 0.02 °C (and 4.85 ± 0.02 °C under excessive light) as compared to the control leaves. 

**Figure 5:**
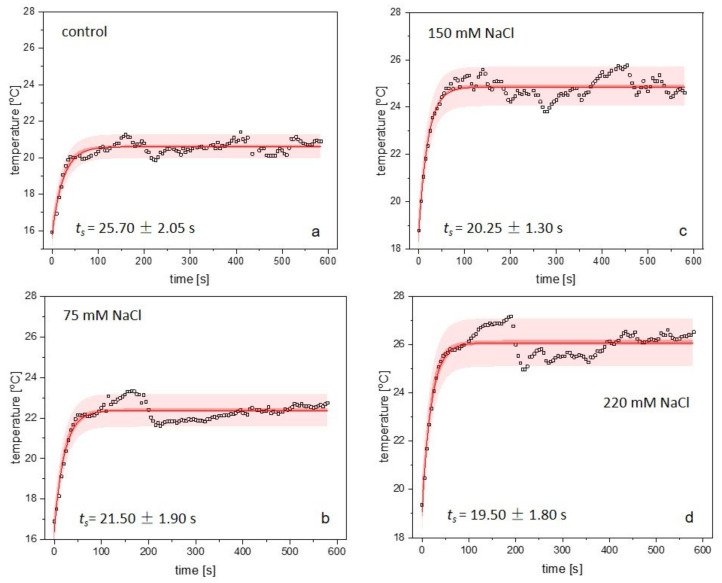
Changes in the temperature of rosette leaves induced by an acute HL treatment measured in *A. thaliana* plants exposed to salinity. Each point on the graph represents the mean value of at least three randomly chosen spots on the thermal image for control (**a**), 75 (**b**), 150 (**c**), and 220 mM (**d**) NaCl treatments. The thermal kinetics were fitted using a mono-exponential function, *y* = *y*_0_ + *y_s_*·[1 − exp(−*t/t_s_*)]. Theoretically determined courses approximate the initial responses of plants to excess light three days after exposure to salinity. Each graph (**a**–**d**) shows the fitted thermal data along with 95% prediction (*light red surfaces*) and confidence (*dark red surfaces*) bands. The light intensity was 2000 µmol·m^−2^·s^−1^.

To show the temporal response of stomatal movement, time-series TGs were used to evaluate the kinetics of temperature changes in plants exposed to acute HL. The data showed that the gradual rise in temperature in the control and salt-treated plants was caused by excessive illumination. The thermal kinetics of the initial response of plants to HL are presented in Figure 5. Each thermal kinetics evaluated for 0 (Figure 5a), 75 (Figure 5b), 150 (Figure 5c), and 220 mM NaCl (Figure 5d) revealed an exponential growth and showed the significant differences in temperature courses between plants that were exposed to different levels of NaCl concentration.

**Figure 6:**
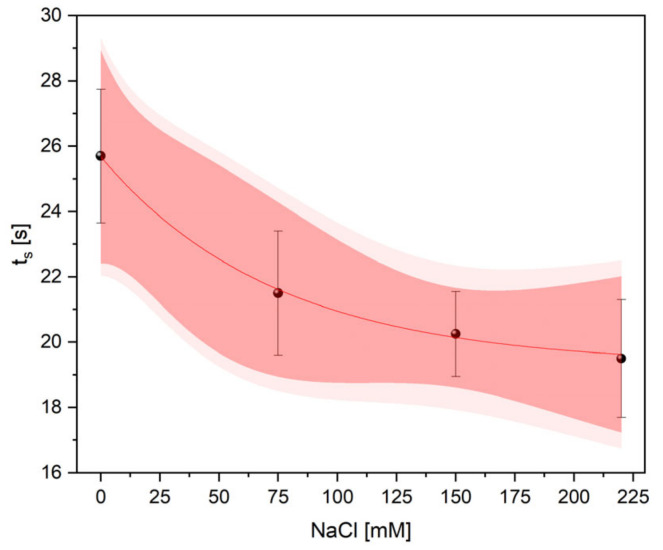
An exponential decay of thermal time constant estimated for control and salt-treated plants as a result of an acute HL treatment. Data are shown along with 95% prediction (*light red surfaces*) and confidence (*dark red surfaces*) bands.

The experimental data, which show temperature changes over time, were fitted using a mono-exponential function *y* = *y*_0_ + *y_s_*·[1 − exp(−*t/t_s_*)], where *y_0_* and *y_s_* is an amplitude and *t_s_* represents the thermal time constant. The bands (Figure 5a–d) represent 95% confidence (dark red) and prediction (light red) intervals. Analysis of thermal rise times revealed that *t_s_* decreased with increasing NaCl concentration (control: *t_s_* = 25.70 ± 2.05 s; 75 mM NaCl: *t_s_* = 21.50 ± 1.90 s; 150 mM NaCl: *t_s_* = 20.25 ± 1.30 s and 220 mM NaCl: *t_s_* = 19.50 ± 1.80 s). The values of *t_s_*, which were determined for non- and salt-treated plants under HL, are presented in (Figure 6) and show an exponential decay. In the case of the highest NaCl concentration (220 mM), the thermal time constant was reduced by almost 25% as compared to the controls.

Since the stomatal closure in NaCl-treated plants affects the temperature of rosette leaves, salinity contributed to the advanced temperature-time courses as compared to control plants. 

## 3. Discussion

Despite the success of thermometry in diagnosing early water stress or salinity [22,25] there are still few reports showing the use of thermal imaging to study the effects of these stresses in plants under dynamic conditions. The results presented here show the use of thermal imaging to study the effects of salinity on *A. thaliana* rosettes under excessive light. We documented that the influence of acute HL is accompanied by rapid heat shock in plant leaves. Hence, the latter should also be considered among the factors which affect stomatal movement. 

Plant growth is linked directly to photosynthesis, transpiration, stomatal regulation, and ionic absorption. Early responses of plants to water and salt stress are very similar because salts hinder the absorption of water through the root system due to the osmotic effects [29]. Under water deficit, with the stomata closed, transpiration is greatly reduced. Our studies show that water content in the non- and salt-treated plants remained unaffected. The absence of consistent significant changes in leaf water content throughout the experimental period suggests that ion accumulation provides an osmotic driving force for the uptake of water in the short-term. This is in agreement with [32] that salt accumulation contributed more efficiently in decreasing the osmotic potential in all salinity treatments than passive dehydration. 

Stomatal opening is a mechanism for leaf cooling, which means that a decrease in stomatal conductance leads to an increase in leaf temperature. The decrease in transpiration rate and stomatal conductance is likely to be the first plant defense against an increasing concentration of sodium ions [30]. Previous gas exchange studies showed the impact of salinity on stomatal conductance with consequent limitations in the CO_2_ assimilation rate [47]. Salinity can affect photosynthesis by stomatal limitations leading to a decrease in carbon assimilation and plant growth. At higher concentrations of NaCl, the inhibition of photosynthetic activity might be due to the closure of stomata and reduced availability of internal CO_2_ [48]. In this study we observed the decreased net CO_2_ assimilation rate for the highest NaCl concentration applied (220 mM NaCl). Interestingly, for the 75 mM NaCl treatment, an increase in photosynthetic rate compared to the control was noticeable. This ‘stimulating’ effect is consistent with other reports in the literature [49,50,51].This may result from better performance of the oxygen-evolving complex (OEC) based on data that suggests beneficial interaction between Cl^−^ and the Mn_4_O_5_Ca cluster of OEC [52]. The stimulation of plant performance under low NaCl concentrations, and inhibition under higher concentrations, is called hormesis and is well-documented in the literature [53]. Our results may indicate that under our experimental conditions, NaCl treatment had a hormetic effect on *A. thaliana.*

Photosynthetic processes are very sensitive to many kinds of stressors, such as salinity, water stress, excessive light, etc. Chlorophyll fluorescence is widely used for estimating the quantum yield (F_V_/F_M_) of photosynthesis in vivo [54]. The ratio of F_V_/F_M_ is an indicator of the maximal photochemical efficiency of PSII photoinhibition [55]. In our studies, only plants exposed to 220 mM NaCl showed a reduced value of F_V_/F_M_ compared to the controls. However, under acute HL, changes in F_V_/F_M_ in all salt-treated plants, before and after the onset of light, were observed. This is consistent with numerous experimental results showing that strong light is the main factor causing photoinhibition which results in a reduction in PSII photochemical efficiency [56,57,58]. The decline in F_V_/F_M_ is due to the inactivation of PSII reaction centers for photoprotection [59] or may be a mechanism to adjust the efficiency of PSII to photosynthetic flux density [60]. 

In this research, we observed a significant increase in temperature in all salt-treated plants. The significant differences in temperature of plants exposed to NaCl were detected during a short period of salinity exposure, even before HL onset. Such observations are consistent with the results of several other studies [25,61], which showed that stomatal closure in saline-stressed plants induced an increase in canopy foliage temperature. The rapid increase in leaf temperature after HL onset is due to increased incident energy on the whole plant rosettes, resulting in reduced stomatal conductance and limited cooling by transpiration [62]. Our results showed that this effect is enhanced by salinity. Moreover, by using thermal imaging, we documented that influence of the acute HL is accompanied by a rapid heat shock. The initial exponential response to NaCl under HL treatment was significantly different between the plants studied. Evaluations using the mono-exponential function *y* = *y*_0_ + *y_s_*·[1 − exp(−*t/t_s_*)] revealed that the thermal time constant *t_s_* declined exponentially with increasing salinity concentration. These results correspond to those of previous experiments performed on *T. salsuginea* characterized by reduced stomatal conductance and transpiration rate [63]. *T. salsuginea* rosettes revealed an advanced temperature-time course and reduced thermal time constant, which was a result of diminished evapotranspiration. A similar effect, which is observed in plants treated with NaCl, may be attributable to insufficient leaf cooling caused by the reduced opening of the stomata [30]. Clearly, the opposite effect was observable in thermal studies of the *ost1-2* mutant with an inability to regulate the stomata, resulting in enhanced stomatal conductance and transpiration rate [64]. In this mutant, the *ost1* mutation contributed to the slowing down of thermal kinetics and a reduction in the thermal rise time, as compared to the control. Other studies [9,65] showed that time constants determined by fitting experimental data using a mono-exponential function for ivy [65] and tobacco [9] exposed to illumination under dark room conditions were 34.5 ± 1.5 s and 43 s, respectively. Unlike [9,65] we did not use paraffin wax to cover the stomata so as not to interfere with their physiological behaviour. The changes in surface roughness caused by this treatment could also modify the airflow over the lamina. Moreover, covering the stomata inhibits transpiration and leaf cooling, so the use of paraffin wax may result in discrepancies between the values of thermal time constants in various studies. Leaf thickness, its roughness, and geometry differ among the species and affect the thermal and optical properties of the plants. Thermal analysis and data evaluation is also limited to the accuracy of the IR equipment used in experiments [65]. The methodological problems mentioned above may contribute to the discrepancies between the values of time constants in various studies. In our study thermal rise times were evaluated on the basis of IR data of juxtaposed plants; therefore, time constants represent relative values between control and salt-treated plants. 

Salinity might cause spatial heterogeneity of stomatal aperture [30]. Interestingly, under HL treatment, TGs showed variations in temperature kinetics over the leaf lamina that may be attributable to stomatal patchiness [66,67] due to hydraulic interactions within the stomatal net [68]. The fluctuations in temperature observed after light onset can be interpreted as a strategy to maximize photosynthesis and avoid heat stress, under dynamic HL conditions [62].

## 4. Materials and Methods

### 4.1. Plant Material and Growth Conditions

*A. thaliana* used in this study was WT (Columbia-0). Seeds were obtained from the Nottingham Arabidopsis Stock Centre (NASC, London, UK). Plants were grown in a controlled environment chamber FytoScope FS2700 (Photon Systems Instruments, Drasov, Czech Republic) under a light intensity of 150 µmol (photons)·m^−2^·s^−1^ and a temperature of 23 °C. Seedlings were grown in a 16/8 h (day/night) photoperiod and the relative humidity in the chamber was maintained at 65%. Three-week-old seedlings were separated into plastic pots and watered twice a week with tap water. For salt-treatment, 4-week-old WT *A. thaliana* plants were irrigated once per lifetime with the following NaCl solutions: 0, 75, 150, and 220 mM. The total volume of tap water and sodium chloride solution used for irrigation was the same for all treatments. All measurements were performed on whole rosettes of 4-week-old plants, three days after the salt treatment. 

### 4.2. Measurement of Gas Exchange

Gas exchange measurements were carried out on *A. thaliana* rosettes consisting of at least 5 young leaves with a portable open gas exchange system LCpro-SD (ADC BioScientific Ltd., Hoddesdon, UK). A 2.14 cm^2^ Arabidopsis cuvette was used with a mixed Red/Blue LED Light Source Head. Rosette leaves were kept in the cuvette for 5 min to reach a steady-state of photosynthesis before the data was recorded. Measurements were performed in ambient CO_2_ saturated conditions (450 µmol·mol^−1^) at 300 µmol∙s^−1^ of airflow, 50–55% relative humidity within the cuvette, a leaf temperature of 25 °C and an irradiance of 130 µmol (photons)·m^−2^·s^−1^ red light intensity in 5 biological repetitions.

### 4.3. Measurement of Infrared Imaging and HL Treatment

Infrared imaging measurements were performed using a thermal camera (Flir Systems Inc., Wilsonville, OR, USA) with a focal plane array, an uncooled microbolometer with 640 × 480 detector elements, a spectral range of 7.5–14 µm, and an accuracy of ±2%. The spatial resolution of the IR camera was 2.62 mrad with a noise equivalent temperature difference of <30 mK. The IR camera was mounted above the plants perpendicular to the plane of leaf rosettes. The thermal images were detected at a frequency of 50 Hz. They were processed using the software IR-Visualizer (Photon Systems Instruments, Drasov, Czech Republic), which offers analytical functions including point temperatures, color profiles, and a recording option. Infrared measurements were carried out under HL conditions of 2000 µmol (photons)·m^−2^·s^−1^ (illuminator SL 3500 emitting white light, equipped with 180 LEDs (Photon Systems Instruments, Drasov, Czech Republic) for 580 s. The illuminator was fixed to the stand with a holder at a distance of 25 cm from the seedlings. The light intensity was monitored with a high- accuracy of PAR measurements using a quantum meter (Apogee, Logan, UT, USA). To compare the thermal images of LL–growing plants with those under the HL stress, some initial TGs were measured before the HL onset. The temperature was measured from the whole rosettes on the thermo-images. In each image, three salt-treated seedlings were juxtaposed with a non-treated control plant to assess the difference in leaf temperature due to salinity, rather than to determine the absolute leaf temperature. All thermal experiments were conducted with at least 3 repetitions and were used to evaluate the kinetics of temperature changes in the plants under investigation (control, 75 mM, 150 mM, and 220 mM NaCl). Time-series TGs were recorded using the software Visualizer, which enabled the sequential acquisition of images at 5 s intervals for 10 min. For each TG of each of the salt-treated and control plants, taken at the same time, the mean of at least three temperature values was calculated. Thermal analysis was performed for rosette leaves that had similar surface areas and were at the same growth stage. The position of the plants did not change during the experiment. The IR experiment was carried out in dim light conditions (<100 µmol (photons)·m^−2^·s^−1^) at room temperature.

### 4.4. Sodium Content Analysis

Sodium concentration in plant samples was analyzed using inductively coupled plasma optical emission spectrometry (ICP-OES) according to the 11,885 ISO standard. An Optima 7300 dv (Perkin Elmer, Inc., Krakow, Poland) spectrometer was used. An analytical emission spectrum line of 589.592 nm was applied. Quantification was achieved using a 5 point external calibration curve based on 4 standard solutions and blank sample analysis. Multi-elemental standard solutions were obtained from Merck (Darmstadt, Germany). Deionized ultrapure water (18.2 MΩcm) was obtained with a Milli-Q system (Millipore, MA, USA). Before ICP-OES analysis, solid samples of plants were transformed into aqueous solutions. A mineralization process was applied for this purpose. A wet digestion procedure was carried out by the addition of 5 mL of 65% nitric acid (V) in a MULTIWAVE 3000 mineralizer (Anton Paar, Ashland, VA, USA). The microwave program was set at a temperature of 230 °C and a pressure of 30.4 bar at a power of 1200–1600 W. The mineralization process was carried out for 25 min and the cooling down process took 30 min. The digests were diluted with deionized water before being introduced to the ICP-OES. The limit of quantification was set at a level of 0.1 mg/L. Precision (as relative standard deviation, RSD) varied between 15% and 25% and accuracy changed from 30% to 40%, depending on the analyte concentration. The expanded relative measurement uncertainty was estimated as 40% for low concentrations and 25% when the sodium concentration was higher.

### 4.5. Leaf Water Content 

Leaf water content was determined by measuring the rosette mass before (F_w_) and after drying (D_w_) in an oven at 105 °C for 1.5 h. Water content (WC) was calculated according to the following Equation (1):

WC(%) = 100·(F_w_ − D_w_)/F_w_, (1)

where F_w_ and D_w_ mean a fresh and a dry mass, respectively [69].

### 4.6. Measurements of Photosynthetic Pigments

The content of photosynthetic pigments was determined spectrophotometrically. Briefly, plant tissue (100 mg fresh mass) was homogenized in 1.5 mL of 80% methanol. Samples were centrifuged for 5 min at 9000× *g* and the supernatant was collected. Absorption spectra of pigment extracts were measured at 470 nm (carotenoids) and 652 and 665 nm (chlorophyll) using a Cary 50 UV-Visible spectrophotometer (Varian, Inc., Palo Alto, CA, USA). The concentrations of Chl and Car were determined according to Lichtenthaler [70].

### 4.7. Measurements of Maximum Quantum Yield of Photosystem II (Maximal Efficiency of PSII Photochemistry)

Maximal PSII photochemical efficiency (F_V_/F_M_) was measured using a FluorPen FP100 fluorometer (Photon Systems Instruments, Drasov, Czech Republic). Before the measurement, rosette leaves were dark-adapted for 20 min to determine the minimum level of fluorescence, F_0_. The saturating pulse intensity was 3000 µmol (photons)·m^−2^·s^−1^ and the measuring light intensity 0.05 µmol (photons)·m^−2^·s^−1^. Excitation light was provided by blue (455 nm) light-emitting diodes. The F_V_/F_M_ in the dark-adapted state was calculated according to the following Equation (2):

F_V_/F_M_ = (F_M_ − F_0_)/F_M_, (2)

where F_M_ is the maximal chlorophyll fluorescence level under dark conditions, F_0_ is the initial chlorophyll fluorescence, and F_V_ is the variable fluorescence [54].

### 4.8. Statistical Analysis and Data Evaluation

The statistics were analyzed and the data evaluated using Origin Professional software version 2019b (Origin-Lab; Northampton, MA, USA). Statistically significant differences between non-and salt-treated plants were determined using ANOVA or the Mann-Whitney *U* test depending on the number of populations considered in the analysis.

## 5. Conclusions

The use of IR thermal imaging as a sensitive and non-invasive method for measuring the temperature of leaves has been discussed in many reports [9,13,15,19]. In the present study the series TGs provided a time-resolved plant response to salinity under acute HL treatment. This allowed us to determine time constants for the increase in leaf temperature caused by a sudden onset of HL. The results presented here show strong evidence that thermal imaging can be used to study the effects of salinity in plants under a dynamic light environment. Other studies have used IR thermal imaging to screen plants exposed to illumination [62,71] but, to the best of our knowledge, there are no reports in the literature on the application of IR thermal kinetics for studying salt-treated plants under HL stress. This technique, particularly the use of time-series TGs, provides information on the kinetics of HL-induced temperature rise in plants exposed to salinity and can be helpful in determining the efficiency of stomatal regulation under short-term salt stress. Our study shows that HL stress is accompanied by rapid heat shock in plant leaves, so the latter should be considered to be one of the factors that affect stomatal movement.

## Data Availability

The data presented in this study are available in article.

## Abbreviations

ABA	abscisic acid
*A. thaliana*	*Arabidopsis thaliana*
Chl *a*	chlorophyll *a*
Car	Carotenoid
F_0_(F_M_)	the minimum (maximum) chlorophyll *a* fluorescence in the dark-adapted state
HL	high light
ICP	inductively coupled plasma emission spectrometry
IR	Infrared
LED	light-emitting diode
LL	low light
PAR	photosynthetically active radiation
PSII	photosystem II
ROS	reactive oxygen species
TG	Thermogram
